# MicroRNA‐539 functions as a tumour suppressor in prostate cancer *via* the TGF‐β/Smad4 signalling pathway by down‐regulating DLX1

**DOI:** 10.1111/jcmm.14402

**Published:** 2019-07-12

**Authors:** Baogang Sun, Yingying Fan, Aijun Yang, Lunan Liang, Jinghe Cao

**Affiliations:** ^1^ Department of Reproductive Medicine Affiliated Hospital of Jining Medical University Jining P.R. China; ^2^ Bidding Office, Affiliated Hospital of Jining Medical University Jining P.R. China

**Keywords:** Distal‐less 1, Epithelial‐mesenchymal transition, invasion, metastasis, MicroRNA‐539, prostate cancer, transforming growth factor β/Smad4 signalling pathway

## Abstract

Prostate cancer (PCa) is the second leading cause of cancer‐related death in males, primarily due to its metastatic potential. The present study aims to identify the expression of microRNA‐539 (miR‐539) in PCa and further investigate its functional relevance in PCa progression both in vitro and in vivo. Initially, microarray analysis was conducted to obtain the differentially expressed gene candidates and the regulatory miRNAs, after which the possible interaction between the two was determined. Next, ectopic expression and knock‐down of the levels of miR‐539 were performed in PCa cells to identify the functional role of miR‐539 in PCa pathogenesis, followed by the measurement of E‐cadherin, vimentin, Smad4, c‐Myc, Snail1 and SLUG expression, as well as proliferation, migration and invasion of PCa cells. Finally, tumour growth was evaluated in nude mice through in vivo experiments. The results found that miR‐539 was down‐regulated and DLX1 was up‐regulated in PCa tissues and cells. miR‐539 was also found to target and negatively regulate DLX1 expression, which resulted in the inhibition of the TGF‐β/Smad4 signalling pathway. Moreover, the up‐regulation of miR‐539 or DLX1 gene silencing led to the inhibition of PCa cell proliferation, migration, invasion, EMT and tumour growth, accompanied by increased E‐cadherin expression and decreased expression of vimentin, Smad4, c‐Myc, Snail1 and SLUG. In conclusion, the overexpression of miR‐539‐mediated DLX1 inhibition could potentially impede EMT, proliferation, migration and invasion of PCa cells through the blockade of the TGF‐β/Smad4 signalling pathway, highlighting a potential miR‐539/DLX1/TGF‐β/Smad4 regulatory axis in the treatment of PCa.

## 1 INTRODUCTION

Prostate cancer (PCa) ranks as the second most frequently diagnosed cancer in male and accounts for approximately 258,000 deaths annually.^1^ The estimated incidence of PCa is about 1/10 000 and the 5‐year survival rate is approximately 72.6%.^2^ PCa falls under the category of epithelial cancers, with an intrinsic potential of metastasizing to distant organs.^3^ Regardless of the remarkable advances made in the treatment of localized malignancies, PCa remains to be incurable due to its high metastatic potential, with most PCa‐related deaths being attributed to the development of metastasis and high resistance to the existing therapies.^4^ Epithelial‐mesenchymal transition (EMT) is closely related to the process of PCa metastasis; therefore, identifying agents that can effectively suppress EMT might be a candidate therapeutic option for metastatic PCa.^5^ A number of microRNAs (miRNAs), including miR‐29b,^6^ miR‐34a,^7^ miR‐21,^8^ and miR‐221 9 have been widely reported to regulate the development of PCa cells. Therefore, there has been a great deal of importance placed on identifying an underlying miRNA that can be a promising biomarker in order to provide an early detection and control of metastasis that occur from PCa.

miRNAs play a major role in the pathogenesis and development of multiple cancers, which is due to their participation in cell differentiation and homoeostasis, as well as their involvement in complex regulatory networks commonly with transcription factors used as their direct targets.^10^ There has been a close correlation detected between some miRNAs including miR‐655, miR‐130b and miR‐590 with the EMT process during cancer progression.^11^, ^12^, ^13^ Moreover, it has been reported that miR‐539 can suppress EMT in oesophageal cancer.^14^ The restoration of miR‐539 has been proven to slow the progression of the proliferation and metastasis abilities of PCa cells by down‐regulating sperm‐associated antigen 5 (SPAG5).^15^ Distal‐less 1 (DLX) was initially discovered in *Drosophila melanogaster*, during which time it was found to regulate the development of nerves and embryo.^16^ Previous studies have demonstrated that the depletion of DLX1 expression could cause PCa cell growth arrest, indicating that DLX1 could be a potential diagnostic target for PCa.^17^ Furthermore, accumulating evidence shows that DLX1 could participate in the biological processes of PCa.^18^ In addition, there is a correlation between the induction of EMT process and the transforming growth factor‐β (TGF‐β) and Smads.^19^ The tumour suppressor Smad4, which has been identified as a pivotal transcription factor in the TGF‐β signalling pathway, has been found to be mutated or deleted frequently in PCa.^20^ Therefore, on the basis of the existing data mentioned above, we conducted the following study to confirm the potential mechanism of miR‐539 in PCa by targeting DLX1 and to determine whether the TGF‐β/Smad4 signalling pathway is involved in the regulation of EMT and metastasis of PCa.

## 2 MATERIALS AND METHODS

### 2.1 Ethics statement

The experiments conducted in the present study have been approved by the Ethics Committee of the Affiliated Hospital of Jining Medical University and signed informed consents were obtained from all participants prior to the study. All animal experiments were conducted in strict accordance with the National Institutes of Health Guide for the Care and Use of Laboratory Animals (National Institutes of Health, Bethesda, MA, USA).

### 2.2 Microarray‐based gene expression profiling

Gene expression datasets related to PCa including GSE55945, GSE45016 and GSE38241 were retrieved from Gene Expression Omnibus (GEO) database (https://www.ncbi.nlm.nih.gov/geo/). Differential analysis of these datasets was conducted using the ‘limma’ package in the R Language Programming with |logFC|> 2 and *P* value < 0.05 as the screening threshold. Next, the expression heatmap of the differentially expressed genes was constructed using the ‘pheatmap’ in the R Language Programming. The Venn diagram of the genes with differential expression retrieved from the chips was constructed using the Venn diagram website (http://bioinformatics.psb.ugent.be/webtools/Venn/) and the results were then intersected.

As a convenient and interactive database for analysis of transcriptome in cancers, UALCAN (http://ualcan.path.uab.edu/index.html), which could be used to retrieve the expression of specific genes in Cancer Genome Atlas (TCGA) database, was used to retrieve the expression of DLX1 in PCa in the present study.

miRNAs regulating DLX1 were predicted by means of microRNA.org (http://34.236.212.39/microrna/home.do), TargetScan (http://www.targetscan.org/vert_71/), mirDIP (http://ophid.utoronto.ca/mirDIP/index.jsp#r) and starBase (http://starbase.sysu.edu.cn/) databases. Next, the intersection was determined for the first 50% miRNAs predicted from the microRNA.org and starBase databases as well as the first 200 miRNAs predicted from the TargetScan and mirDIP databases. Afterwards, the Venn diagram was constructed using the Venn diagram website and the intersection of the miRNAs predicted from these four databases was obtained. The mirSVR score of the miRNA regulating DLX1 predicted from the microRNA.org database was analysed to further determine the candidate miRNA.

### 2.3 Study subjects

The PCa tissues and adjacent normal tissues were obtained from 134 patients in the Affiliated Hospital of Jining Medical University from January 2015 to December 2017 for the current study. The mean age of all patients was 63.4 ± 7.9 years old, including 36 patients under the age of 60 years and 98 patients over the age or equal to 60 years. Out of the above patients, there were 71 patients with family history of PCa and the remaining 63 patients had no family history of PCa. A total of 64 patients had presented with lymph node metastasis while 70 patients did not have lymph node metastasis. According to Gleason score, 88 patients had the score <7 and 46 patients had the score ≥7. The inclusion criteria when selecting patients for the present study were as follows 21: (a) the diagnosis of PCa had been pathologically confirmed; (b) the patients had not received any anticancer treatment including radiotherapy, chemotherapy and immunotherapy prior to surgery; (c) patients had undergone transrectal prostate puncture; (d) complete clinical, pathological and prognostic records were accessible. The exclusion criteria were as follows^21^: (a) patients who had non‐prostate cancer metastasis in other parts of the body; (b) patients who died from other cancers; (c) patients with incomplete relevant records. In addition, all patients included patients in this study had no immunological or connective tissue diseases and had no comorbidities of vital organs.

### 2.4 Immunohistochemistry

Paraffin‐embedded specimens were cut into 4‐μm thick sections after being fixed in 10% formaldehyde and placed in a 60°C oven for l hour. The sections were dewaxed routinely with xylene, dehydrated with graded alcohol and incubated in 3% H_2_O_2_ (Sigma‐Aldrich Corp., St. Louis, MO, USA) at 37°C for 30 minutes. After being rinsed with phosphate buffered saline (PBS), the sections were boiled in 0.01 M citrate buffer for 20 minutes at 95°C. After being cooled down to room temperature, the sections were sealed with normal goat serum at 37°C for 10 minutes, followed by incubation at 4°C overnight with the addition of primary antibody mouse polyclonal antibody to DLX1 (50 μL) (ab167575, 1:50, Abcam Inc, Cambridge, UK). Subsequently, the sections were incubated with the biotin‐labelled goat polyclonal secondary antibody to mouse immunoglobulin G (IgG) (ab6789, 1:1000, Abcam Inc, Cambridge, MA, USA) at 37°C for 30 minutes. Then, the sections were developed by diaminobenzidine (DAB) (Sigma‐Aldrich Corp., St. Louis, MO, USA). Five high‐fold fields (100 cells in each field) were randomly selected from each section and photographed, after which the positive cells were counted and the positive rate was calculated.

### 2.5 Dual luciferase reporter gene assay

The target gene of miR‐539 was conducted using the microRNA.org. Dual luciferase reporter gene assay was conducted to confirm whether DLX1 was the target gene of miR‐539. The 3′‐untranslated region (3′UTR) of the DLX1 gene was cloned into pmirGLO vector (E1330, Promega Corp., Madison, WI, USA) and was given the name pDLX1 wild‐type (Wt). Site‐directed mutagenesis was performed on the potential binding sites of miR‐539 and the pDLX1‐mutant (Mut) vector was also constructed. pRL‐TK vector expressing Renilla luciferase (E2241, Promega Corp., Madison, WI, USA) was constructed to serve as an internal control. The miR‐539 mimic or the NC plasmids were transfected into cells together with pDLX1‐Wt or pDLX1‐Mut. After 48 hours of transfection, the cells were collected and luciferase activity was detected according to the instructions of the luciferase assay kit (GeneCopoeia, Inc, Rockville, Maryland, USA).

### 2.6 Cell culture and grouping

Cell lines PC3 and DU145 (Shanghai Institute of Biochemistry and Cell Biology Chinese Academy of Sciences, Shanghai, China) were added with the Dulbecco's modified Eagle medium (DMEM) conjugated with 10% foetal bovine serum (FBS) and incubated with 5% CO_2 _at 37°C. Next, the cells were classified into blank (PCa cells without transfection), negative control (NC) (PCa cells transfected with miR‐539 NC), miR‐539 mimic (PCa cells transfected with miR‐539 mimic), miR‐539 inhibitor (PCa cells transfected with miR‐539 inhibitor), si‐DLX1 (PCa cells transfected with si‐DLX1), miR‐539 inhibitor + si‐DLX1 (PCa cells transfected with miR‐539 inhibitor and si‐DLX1), SB‐431542 (DU145 cells transfected with TGF‐β signalling pathway inhibitor SB‐431542 for 1.5 hours with the concentration of 10 µM), DMSO (DU145 cells transfected with 10 µM DMSO, taken as the control of SB‐431542), miR‐539 inhibitor + DMSO (DU145 cells transfected with miR‐539 inhibitor and DMSO) and miR‐539 inhibitor + SB‐431542 (DU145 cells transfected with miR‐539 inhibitor and SB‐431542) groups. The aforementioned plasmids had all been purchased from Sangon Biotech Co., Ltd. (Shanghai, China). The transfection was performed according to the instructions of lipofectamine 2000 (11668‐019, Invitrogen Inc, Carlsbad, CA, USA).

### 2.7 Reverse transcription quantitative polymerase chain reaction (RT‐qPCR)

Levels of DLX1 and downstream target genes of TGF signalling pathway were determined using RT‐qPCR. The primers for RT‐qPCR are listed in Table 1. The cells were then collected following transfection, after which the total RNA was extracted with the use of a Trizol kit (Invitrogen Inc, Carlsbad, CA, USA) and reverse transcribed into cDNA. The qPCR was performed with cDNA used as the template under the following conditions: 95°C for 5 minutes (1 cycle), 45 cycles of 95°C for 20 seconds, 60°C for 1 minute and 72°C for 30 second. β‐Actin and U6 served as internal references. The fold changes were calculated by means of relative quantification (2^−ΔΔCT^ method), using the following formula: ΔCt = Ct _target gene_‐Ct _internal control gene _and ΔΔCT = ΔCt _experimental group_‐ΔCt _control group_.

**Table 1:** Primer sequences of related genes for reverse transcription quantitative polymerase chain reaction

Gene	Primer sequence (5′‐3′)
β‐actin	Forward: CATATGGCTACATAGGATGATGATATCCCCC
	Reverse: GAATTCACAGAATTCCTAGAAGCATTTGCGGTG
DLX1	Forward: GCGGCCTCTTTGGGACTCACA
	Reverse: GGCCAACGCACTACCCTCCAGA
miR‐539	Forward: GAAGAGGCTAACGTGAGGTTG
	Reverse: CACCATGACCAAGCCACGTAG
c‐myc	Forward: ATGCCCTCAACGTTAGCTTCACC
	Reverse: TTACGCACAAGAGTTCCGTAGCTGTTC
E‐cadherin	Forward: AACGCATTGCCACATACA
	Reverse: GAGCACCTTCCATGACGAC
vimentin	Forward: ACAGGCTTTAGCGAGTTATT
	Reverse: GGGCTCCTAGCGGTTTAG
Smad4	Forward: GGACCGGATTACCCAAGACACA
	Reverse: CTGCAATCGGCATGGTATGAAG
Snail1	Forward: TTCCAGCGCCCTACGACCAG
	Reverse: GCCTTTCCCACTGTCCTCATC
SLUG	Forward: CCTTCCTGGTCAAGAAGCATTTCA
	Reverse: AGGCTCACATATTCCTTGTCACAG
U6	Forward: CTCGCTTCGGCAGCACA
	Reverse: AACGCTTCACGAATTTGCGT

Abbreviations: DLX1, distal‐less homeobox 1; miR‐539, microRNA 539.

### 2.8 Western blot analysis

Once the total protein had been extracted, the protein concentration was measured using the bicinchoninic acid (BCA) Protein Assay Kit (20201ES76, Yeasen Biotech Co., Ltd., Shanghai, China). After separation with 8% sodium dodecyl sulphate‐polyacrylamide gel electrophoresis (SDS‐PAGE), the protein was transferred onto polyvinylidene fluoride (PVDF) membrane. Afterwards, the membrane was blocked with 5% skimmed milk and incubation was carried out at 4°C overnight with the addition of the following primary antibodies: mouse monoclonal antibody to β‐actin (ab8226, 1:2000, Abcam Inc, Cambridge, UK), rabbit polyclonal antibody to DLX1 (ab126054, 1:500, Abcam Inc, Cambridge, MA, UK), rabbit monoclonal antibody to c‐Myc (ab32072, 1:1000, Abcam Inc, Cambridge, UK), mouse monoclonal antibody to E‐cadherin (ab1416, 1:100, Abcam Inc, Cambridge, UK), vimentin (ab92547, 1:1500, Abcam Inc, Cambridge, UK), Smad4 (ab40759, 1:2000, Abcam Inc, Cambridge, UK), rabbit polyclonal antibody to Snail1 (ab82846, 1:500, Abcam Inc, Cambridge, UK) and rabbit polyclonal antibody to SLUG (ab27568, 1:500, Abcam Inc, Cambridge, UK). After receiving three washes with PBS supplemented with 0.05% phosphate buffered saline Tween 20 (PBST), the membrane was incubated with horseradish peroxidase‐labelled goat antimouse IgG secondary antibody (ab6789, 1:2000, Abcam Inc, Cambridge, UK) or ready‐to‐use goat anti‐rabbit IgG secondary antibody (ab6721, 1:2000, Abcam Inc, Cambridge, UK) at room temperature for 1 hour. Next, the membrane was rinsed three times with PBST and exposed using enhanced chemiluminescence (ECL) solution (ECL808‐25, Biomiga. Inc, San Diego, CA, USA) under dark conditions. Relative protein levels of the target genes were expressed as the ratio of grey values of the target protein bands to those of the β‐actin band.

### 2.9 3‐(4,5‐dimethylthiazol‐2‐yl)‐2,5‐diphenyltetrazolium bromide (MTT) assay

The cells in the logarithmic growth stage were inoculated into 96‐well plates at a density of 2.5 × 10^5^ cells/mL. After 24, 48 and 72 hours, the cells in each well were incubated with 10 μL MTT solution (5 mg/mL) (Sigma‐Aldrich Corp., St. Louis, MO, USA). Subsequently, the plates were further cultured for 4 hours. An automated reader (BIO‐RAD Inc, Hercules, CA, USA) was employed to detect the optical density (OD) values of each well at 490 nm. The experiment was repeated in triplicate.

### 2.10 Scratch test

After 48 hours of treatment, the cells were seeded into 6‐well plates. Upon reaching full adherence, the cells were cultured in serum‐free DMEM culture medium. When the cell confluence reached 90%‐100%, the cells were scratched slowly with a sterile pipette tip (10 μL) perpendicular to the plate bottom. Each well was expected to have 4‐5 scratches with the same width. Next, the plates received three washes with PBS, followed by cell collection. An inverted microscope was used to measure the metastasis distance at 0 hour and 24 hours following scratching with various randomly selected visual fields. The photographs were obtained and the experiment was conducted in triplicate.

### 2.11 Transwell assay

The 8‐μm Transwell chambers (Corning Inc, Corning, NY, USA) with 24 wells were applied. The chamber was coated with 50 μL Matrigel (Sigma‐Aldrich Corp., St. Louis, MO, USA). Cells (200 μL) were seeded into the apical chamber and complete medium (300 μL, Invitrogen Inc, Carlsbad, CA, USA) conjugated with 10% FBS was added into the basolateral chamber. The chambers were then incubated at 37°C with 5% CO_2_. After a 48‐h incubation, the cells were fixed and stained with 0.5% crystal violet. An inverted microscope (XDS‐800D, Shanghai Caikon Optical Instrument Co., Ltd., Shanghai, China) was used to count the stained cells. PCa cell invasion was determined using the average number of cells in five randomly selected fields.

### 2.12 Tumour formation in nude mice

The single cell suspension was prepared using PCa cell lines PC3 and DU145 respectively. The PBS and Matrigel (E1270, Sigma‐Aldrich Corp., St. Louis, MO, USA) were mixed at the ratio of 1:1 and cells were suspended at the final concentration of 1 × 10^6^ cells/200 μL. A total of 72 nude mice (SLAC Laboratory Animal Co. Ltd, Shanghai, China) were categorized into blank, NC, miR‐539 mimic, miR‐539 inhibitor, siRNA‐DLX1 and miR‐539 inhibitor + siRNA‐DLX1 groups (n = 6 in each group). After being anesthetized with ether, the mice in each group were inoculated subcutaneously with transfected PC3 and DU145 cells (1 × 10^6^ cells/200 μL) in the left armpit respectively. The mice were then raised under the same environment and observed every 7 days beginning from the 7th day. Tumour length and width were recorded and the gross tumour volume was calculated according to the formula, volume = (length×width^2^)/2. On the 35th day, the nude mice were killed, followed by tumour resection. Six tumours were weighed in each group.

### 2.13 Statistical analysis

Statistical analysis was conducted by SPSS 19.0 (IBM Corp. Armonk, NY, USA). Measurement data were presented as mean ± standard deviation. Data between two groups were compared using independent‐sample *t* test and data among multiple groups were compared using one‐way analysis of variance (ANOVA). Fisher's least significant difference (LSD) method was adopted for pairwise comparison. Enumeration data were presented as percentage and analysed using chi‐square test. A value of *P* < 0.05 indicated statistical significance.

## 3 RESULTS

### 3.1 miR‐539 participates in the development of PCa by regulating DLX1

**Figure 1:**
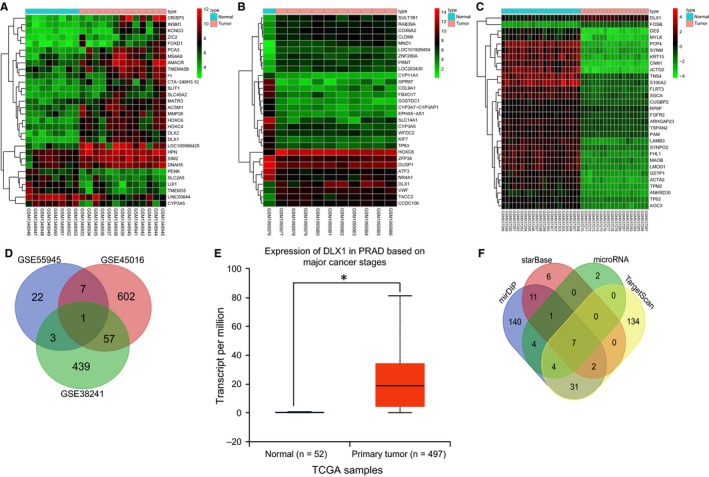
Microarray‐based gene expression profiling identifies the involvement of hsa‐miR‐539 in the development of PCa by mediating DLX1. A‐C, expression heatmap of PCa‐related gene expression profile, in which the abscissa refers to sample number, the ordinate refers to gene name and the upper crossband refers to sample type. Blue represents normal control sample and red represents PCa sample. The right histogram represents colour grade, in which red indicates high expression and green indicates poor expression. The upper dendrogram refers to sample cluster and the left dendrogram refers to gene expression cluster. Each circle represents the expression of a gene in one sample; D, Venn analysis of differentially expressed genes from three datasets, in which blue refers to the number of differentially expressed genes in GSE55945 datasets, red refers to the number of differentially expressed genes in GSE45016 datasets and green refers to the number of differentially expressed genes in GSE38241 datasets. The middle part is the intersection of three datasets and the number in the image is the number of differentially expressed genes in each region; E, expression of DLX1 in PCa samples and normal control samples from the TCGA database, in which the abscissa represents sample cluster, the ordinate represents the expression of DLX1 gene, the left blue box plot refers to the expression of DLX1 in normal control samples and the right red box plot refers to the expression of DLX1 gene in PCa samples; F, the predicted miRNAs regulating DLX1 gene, in which blue represents the miRNAs predicted from the mirDIP database, red represents the miRNAs predicted from the starBase database, green refers to the miRNAs predicted from the microRNA.org database and yellow refers to the miRNAs predicted from the TargetScan database. The region with number 7 is the intersection of the prediction results from four databases, suggesting that there are seven miRNAs existing simultaneously in these four databases. *P < 0.05, compared with primary tumour group or normal group; PCa, prostate cancer; miR‐539, microRNA‐539; TGF‐β, transforming growth factor‐β; DLX1, distal‐less homeobox 1; TCGA, The Cancer Genome Atlas

Through differential analysis, 33, 667 and 1008 differentially expressed genes were obtained from the GSE55945, GSE45016 and GSE38241 datasets which were retrieved from the GEO database respectively. Next, the expression heatmap of 30 differentially expressed genes was constructed (Figure 1A‐C). In order to screen out the genes associated with PCa, Venn analysis was conducted to analyse all the differentially expressed genes in GSE55945 and GSE45016 datasets as well as the first 500 significantly differentially expressed genes in the GSE38241 dataset (Figure 1D), the results of which showed that DLX1 was the only one of them that was simultaneously present and up‐regulated in all the three datasets. Then the expression of DLX1 in PCa samples and normal control samples from the TCGA database was analysed and the results revealed that there was a high expression in DLX1 in PCa samples (Figure 1E). It has been reported that DLX1 could influence disease development *via* the TGF‐β/Smad4 signalling pathway^22^, ^23^ and the TGF‐β/Smad4 signalling pathway was previously found to be associated with PCa.^24^, ^25^ Hence, there is a high possibility that DLX1 could affect the development of PCa through the TGF‐β/Smad4 signalling pathway. In order to figure out the potential miRNAs regulating DLX1, the top 50% miRNAs were predicted from the microRNA.org and starBase databases and top 200 miRNAs predicted from the mirDIP and TargetScan databases were subjected to Venn analysis. The results displayed that there were seven miRNAs in the intersection of the prediction results from four databases (Figure 1F). The binding abilities of these seven miRNAs to DLX1 were scored <−0.6 (Table 2). Therefore, miR‐539 was selected for subsequent studies and we investigated whether miR‐539 could influence the development of PCa through the TGF‐β/Smad4 signalling pathway by mediating DLX1.

**Table 2:** Binding of miRNAs to DLX1

miRNA	mirSVR score	PhastCons score
hsa‐miR‐543	−1.3055	0.7483
hsa‐miR‐19a	−1.1034	0.7478
hsa‐miR‐19b	−1.1034	0.7478
hsa‐miR‐539	−0.7944	0.5816
hsa‐miR‐320a	−0.2288	0.5639
hsa‐miR‐320b	−0.2288	0.5639
hsa‐miR‐320c	−0.193	0.5631

Note

miRNA refers to the predicted miRNAs regulating DLX1; mirSVR score is thermodynamic stability score (the criterion is ≤−0.1) and the lower the score, the stronger of the binding ability of miRNA‐mRNA, suggesting a higher possibility that miRNA down‐regulates genes; PhastCons score represents the evolutionary conservatism of gene untranslated region in species and stronger evolutionary conservatism reflects better prediction; miR, microRNA; DLX1, distal‐less homeobox 1.

### 3.2 PCa tissues presented with a high expression of DLX1

**Figure 2:**
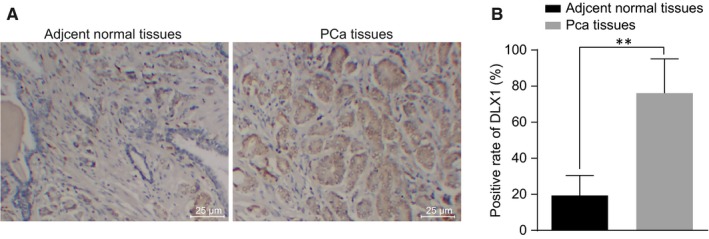
The positive expression of DLX1 protein is increased in PCa tissues. A, immunohistochemical staining analysis of DLX1 protein in the PCa tissues and adjacent normal tissues; B, quantitative analysis for the positive expression rate of DLX1 protein in the PCa tissues and adjacent normal tissues; **P < 0.01, compared with PCa tissues or adjacent normal tissues; PCa, prostate cancer; DLX1, distal‐less homeobox 1

The results from the immunohistochemistry, which was performed in order to detect the positive expression of DLX1 in PCa, suggested that DLX1 was positively stained brown and located at the cytoplasm (Figure 2A). PCa tissues exhibited a positive rate of DLX1 of 76.92% and the adjacent normal tissues displayed 18.46% (Figure 2B). Compared with the adjacent normal tissues, the protein expression of DLX1 in PCa tissues was considerably increased (*P* < 0.05). These findings indicate that there was a higher positive rate of DLX1 protein expression in the PCa tissues.

### 3.3 PCa tissues exhibit reduced miR‐539 but elevated DLX1

**Figure 3:**
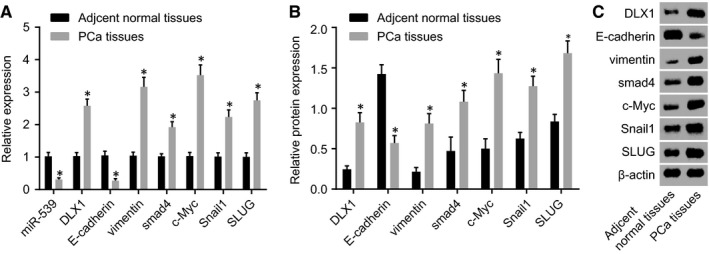
PCa tissues display poor expression of miR‐539 and E‐cadherin yet high expression of DLX1, Smad4, c‐Myc, vimentin, Snail1 and SLUG. A, miR‐539 expression and mRNA expression of DLX1, Smad4, c‐Myc and vimentin, Snail1, SLUG and E‐cadherin in the PCa tissues and the adjacent normal tissues detected by RT‐qPCR; B and C, Western blot analysis of DLX1, Smad4, c‐Myc and vimentin, Snail1, SLUG and E‐cadherin proteins in the PCa tissues and the adjacent normal tissues; *P < 0.05, compared with the adjacent normal tissues; PCa, prostate cancer; miR‐539, microRNA‐539; DLX1, distal‐less homeobox 1; RT‐qPCR, reverse transcription quantitative polymerase chain reaction

RT‐qPCR and Western blot analysis were carried out in order to further identify the expression of miR‐539, DLX1, Smad4, c‐Myc, vimentin, E‐cadherin, Snail1 and SLUG in the PCa tissues. The results (Figure 3) indicated that there was a significant elevation in the expression levels of DLX1, Smad4, c‐Myc, vimentin, Snail1 and SLUG in the PCa tissues than those in adjacent normal tissues (*P* < 0.05). However, the expression levels of miR‐539 and E‐cadherin were greatly reduced in the PCa tissues (*P* < 0.05). These findings provided evidence that miR‐539 level is reduced in PCa tissues.

### 3.4 The expression of DLX1 and miR‐539 is correlated with lymph node metastasis, pathological stage, tumour size and Gleason scores of PCa

The correlation between the clinical characteristics of PCa patients and the expression of DLX1 and miR‐539 was determined. As shown in Table 3, the results found that the expression levels of DLX1 and miR‐539 were not statistically related to age and family history of patients with PCa (*P* > 0.05), but were associated with lymph node metastasis, pathological stages, tumour size and Gleason scores of PCa (*P* < 0.05).

**Table 3:** Correlations of expression levels of DLX1 and miR‐539 with clinicopathological features of the patients with prostate cancer

Clinicopathological features	DLX1 expression		*P*	miR‐539 expression		*P*
	Low	High		Low	High	
Age (year)			0.138			0.274
<60	21	15		16	20	
≥60	43	55		54	44	
Family history			0.706			0.508
No	29	34		31	32	
Yes	35	36		39	32	
Lymph node metastasis			0.023			0.003
No	40	30		28	42	
Yes	24	40		42	22	
Pathological stages			0.036			0.004
High differentiation	29	17		17	29	
Moderate differentiation	13	18		14	17	
Low differentiation	22	35		39	18e	
Tumour size			0.012			0.033
<2 cm	48	38		39	47	
≥2 cm	16	32		31e	17	
Gleason scores			0.001			0.030
<7	51	37		40	48	
≥7	13	33		30	16	

Abbreviations: DLX1, distal‐less homeobox 1; miR‐539, microRNA‐539.

### 3.5 DLX1 is a target gene of miR‐539

**Figure 4:**
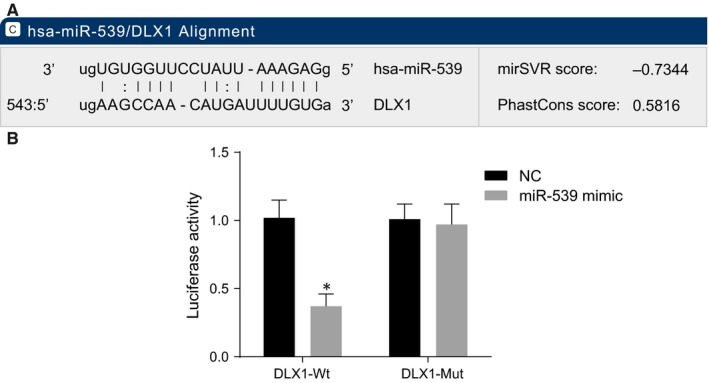
DLX1 is a target gene of miR‐539. A, predicted binding site of miR‐539 on the 3'‐UTR of DLX1; B, luciferase activity in the NC and miR‐539 mimic groups detected by dual luciferase reporter gene assay; *P < 0.05, compared with the NC group; miR‐539, microRNA‐539; DLX1, distal‐less homeobox 1; NC, negative control; 3′‐UTR, 3′‐untranslated region; Wt, wild‐type; Mut, mutant

Since DLX1 was predicted to be targeted by miR‐539 (Figure 4A), dual luciferase reporter gene assay was performed to verify the target relationship between miR‐539 and DLX1. Compared with the NC group, there was a decrease in the luciferase activity of Wt‐miR‐539/DLX1 in the miR‐539 mimic transfection group (*P* < 0.05) (Figure 4B). However, the luciferase activity did not differ greatly in the Mut‐miR‐539/DLX1 transfection group (*P* > 0.05). Therefore, miR‐539 could specifically bind to DLX1 gene.

### 3.6 Up‐regulation of miR‐539 or silencing of DLX1 increases the expression of E‐cadherin and decreases the expression of vimentin, c‐Myc and Smad4 in PCa cells

**Figure 5:**
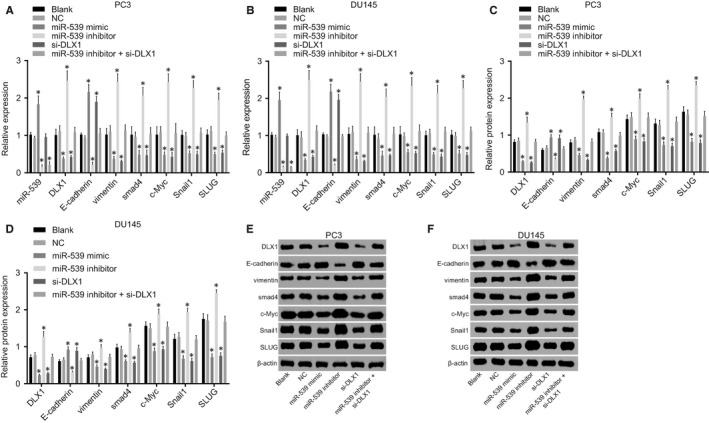
miR‐539 promotes the E‐cadherin expression and inhibits expression of DLX1, vimentin, c‐Myc, Smad4, Snail1 and SLUG. A, mRNA expression of DLX1, vimentin, E‐cadherin, c‐Myc, Smad4, Snail1 and SLUG in PC3 cell lines transfected with miR‐539 mimic, miR‐539 inhibitor, si‐DLX1 and miR‐539 inhibitor + si‐DLX1 detected by RT‐qPCR; B, mRNA expression of DLX1, vimentin, E‐cadherin, c‐Myc, Smad4, Snail1 and SLUG in DU145 cell lines transfected with miR‐539 mimic, miR‐539 inhibitor, si‐DLX1 and miR‐539 inhibitor + si‐DLX1 detected by RT‐qPCR; C and E, Western blot analysis of DLX1, vimentin, E‐cadherin, c‐Myc, Smad4, Snail1 and SLUG proteins in PC3 cell lines transfected with miR‐539 mimic, miR‐539 inhibitor, si‐DLX1 and miR‐539 inhibitor + si‐DLX1; D and F, Western blot analysis of DLX1, vimentin, E‐cadherin, c‐Myc, Smad4, Snail1 and SLUG proteins in DU145 cell lines transfected with miR‐539 mimic, miR‐539 inhibitor, si‐DLX1 and miR‐539 inhibitor + si‐DLX1; *P < 0.05, compared with the blank group and the NC group; miR‐539, microRNA‐539; DLX1, distal‐less homeobox 1; NC, negative control; si, small interfering; RT‐qPCR, reverse transcription quantitative polymerase chain reaction

Next, RT‐qPCR and Western blot analysis (Figure 5) were conducted to determine expression levels of DLX1, vimentin, E‐cadherin, c‐Myc, Smad4, Snail1 and SLUG in PCa cells. It was shown that PC3 and DU145 cell lines presented the same tendency. There was no evident difference in the expression of DLX1, vimentin, c‐Myc, E‐cadherin and Smad4 between the blank and NC groups (*P* > 0.05). Compared with the blank and NC groups, there was a significant decrease in miR‐539 level in the miR‐539 inhibitor and miR‐539 inhibitor + si‐DLX1 groups, while it was increased in the miR‐539 mimic group (*P* < 0.05) and there was no remarkable difference observed in the expression of miR‐539 in the si‐DLX1 group (*P* > 0.05). Moreover, compared with the blank and NC groups, the expression level of E‐cadherin in the miR‐539 mimic and si‐DLX1 groups was up‐regulated (*P* < 0.05) and expression levels of vimentin, c‐Myc, Smad4, Snail1 and SLUG were down‐regulated (*P* < 0.05), while the miR‐539 inhibitor group presented with the opposite results (*P* < 0.05) and there was no significant difference detected in the miR‐539 inhibitor + si‐DLX1 group (*P* > 0.05). These findings suggested that the miR‐539 can promote the expression level of E‐cadherin while inhibiting the expression levels of DLX1, vimentin, c‐Myc and Smad4, Snail1 and SLUG.

### 3.7 Up‐regulation of miR‐539 or silencing of DLX1 inhibits PCa cell proliferation

**Figure 6:**
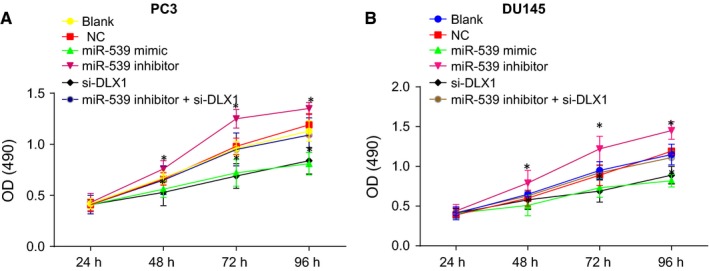
Up‐regulation of miR‐539 or silencing of DLX1 represses PCa cell viability. A, PC3 cell viability in response to miR‐539 mimic, miR‐539 inhibitor, si‐DLX1 and miR‐539 inhibitor + si‐DLX1 measured by MTT assay; B, DU145 cell viability in response to miR‐539 mimic, miR‐539 inhibitor, si‐DLX1 and miR‐539 inhibitor + si‐DLX1 measured by MTT assay; *P < 0.05, compared with the blank group and the NC group; PCa, prostate cancer; miR‐539, microRNA‐539; MTT, 3‐(4,5‐dimethylthiazol‐2‐yl)‐2,5‐diphenyltetrazolium bromide; OD, optical density; NC, negative control; si, small interfering

MTT assay was employed to determine cell viability. The results (shown in Figure 6) elucidated that both PC3 and DU145 cell lines presented with the consistent trend. There was no significant difference observed in the cell proliferation in the blank and NC groups (*P* > 0.05). Compared with the blank and NC groups, the miR‐539 inhibitor group showed distinctly increased cell proliferation, but the miR‐539 mimic and si‐DLX1 groups had evidently inhibited cell proliferation (*P* < 0.05). The miR‐539 inhibitor + si‐DLX1 group had no significant difference (*P* > 0.05). These results suggested that overexpression of miR‐539 or silencing DLX1 could result in the suppression of the proliferation of PCa cells.

### 3.8 Up‐regulation of miR‐539 or silencing of DLX1 suppresses PCa cell migration

**Figure 7:**
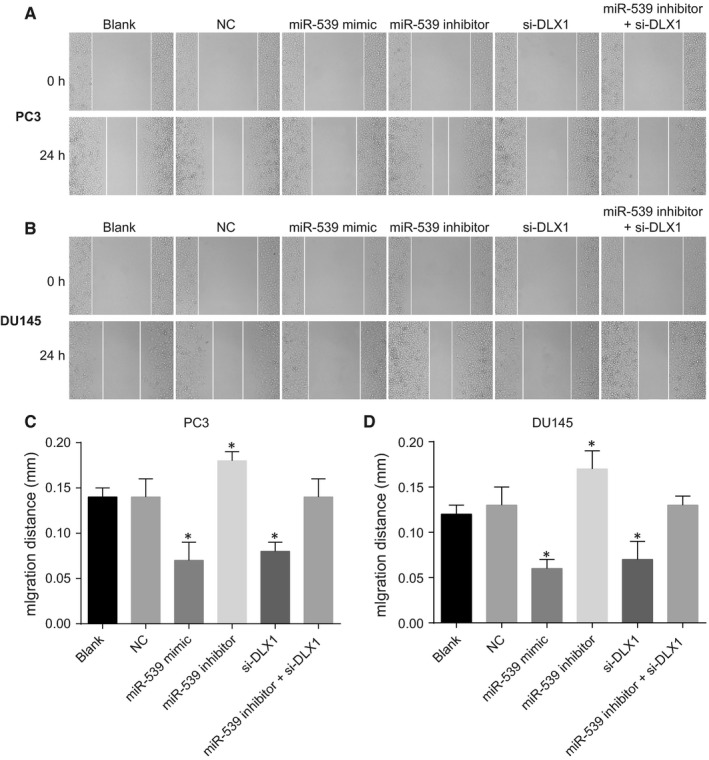
Up‐regulation of miR‐539 or silencing of DLX1 restrains PCa cell migration. A and C, PC3 cell migration and the quantitative analysis in response to miR‐539 mimic, miR‐539 inhibitor, si‐DLX1 and miR‐539 inhibitor + si‐DLX1 measured by scratch test; B and D, DU145 cell migration and the quantitative analysis in response to miR‐539 mimic, miR‐539 inhibitor, si‐DLX1 and miR‐539 inhibitor + si‐DLX1 measured by scratch test; *P < 0.05, compared with the blank group and the NC group; miR‐539, microRNA‐539; PCa, prostate cancer; NC, negative control; si, small interfering

Scratch test was adopted to detect cell migration. As shown in Figure 7, migration of PC3 and DU145 cell lines displayed the consistent trend. The blank and the NC groups had no significant difference concerning PCa cell migration (*P* > 0.05). There was a dramatic decrease in the migration of PCa cells in the miR‐539 mimic group and the si‐DLX1 group in comparison to the blank and NC groups (*P* < 0.05), while it drastically increased in the miR‐539 inhibitor group (*P* < 0.05) and there was no significant difference in the inhibitor + si‐DLX1 group (*P* > 0.05). These results demonstrated that up‐regulation of miR‐539 or DLX1 gene silencing can lead to the suppression of migration of PCa cells.

### 3.9 Up‐regulation of miR‐539 or silencing of DLX1 inhibits PCa cell invasion

**Figure 8:**
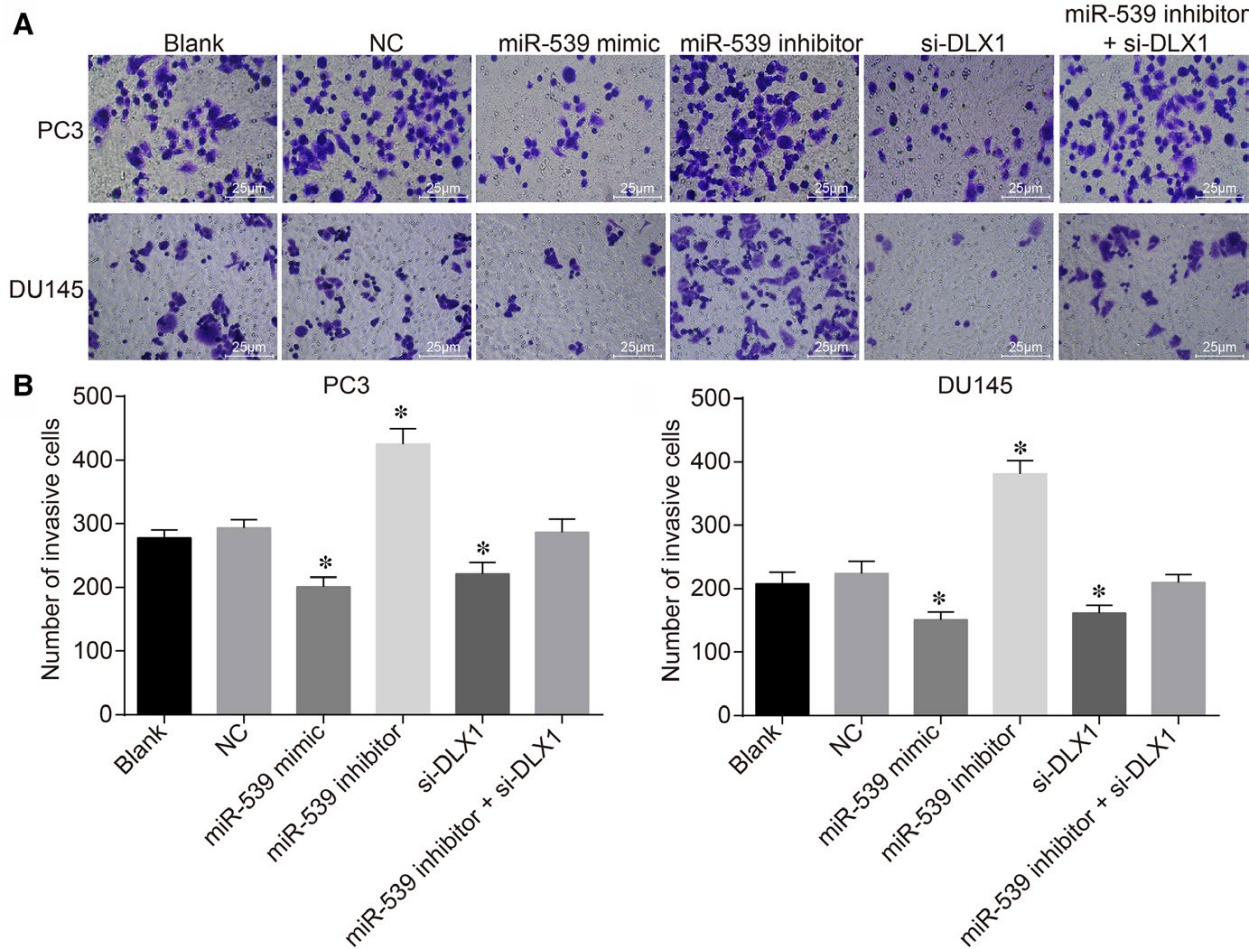
Up‐regulation of miR‐539 or silencing of DLX1 inhibits PCa cell invasion. A, invasion of PC3 cell lines and DU145 cell lines transfected with miR‐539 mimic, miR‐539 inhibitor, si‐DLX1 and miR‐539 inhibitor + si‐DLX1 measured by transwell assay (×200); B, quantitative analysis for the number of invasive PC3 cells transfected with miR‐539 mimic, miR‐539 inhibitor, si‐DLX1 and miR‐539 inhibitor + si‐DLX1 measured by transwell assay; C, quantitative analysis for the number of invasive DU145 cells transfected with miR‐539 mimic, miR‐539 inhibitor, si‐DLX1 and miR‐539 inhibitor + si‐DLX1 measured by transwell assay; *P < 0.05, compared with the blank group and the NC group; miR‐539, microRNA‐539; PCa, prostate cancer; NC, negative control; si, small interfering

The Transwell assay was conducted in order to measure PCa cell invasion following treatment with miR‐539 mimic, miR‐539 inhibitor, si‐DLX1 and miR‐539 inhibitor + si‐DLX1 (Figure 8). The trend of PCa cell invasion in PC3 and DU145 cell lines was consistent. The blank and NC groups showed no significant difference regarding the invasion of PCa cells (*P* > 0.05). Compared with the blank and NC groups, the PCa cell invasion in the miR‐539 mimic and siRNA‐DLX1 groups was evidently reduced, while the miR‐539 inhibitor group showed the opposite trend (*P* < 0.05) and the miR‐539 inhibitor + si‐DLX1 group presented with no obvious difference (*P* > 0.05). The aforementioned results revealed that miR‐539 overexpression or DLX1 gene silencing could result in the suppression in cell invasion in PCa cells.

### 3.10 The expression of DLX1 and miR‐539 is correlated with lymph node metastasis, pathological stage, tumour size and Gleason scores of PCa

**Figure 9:**
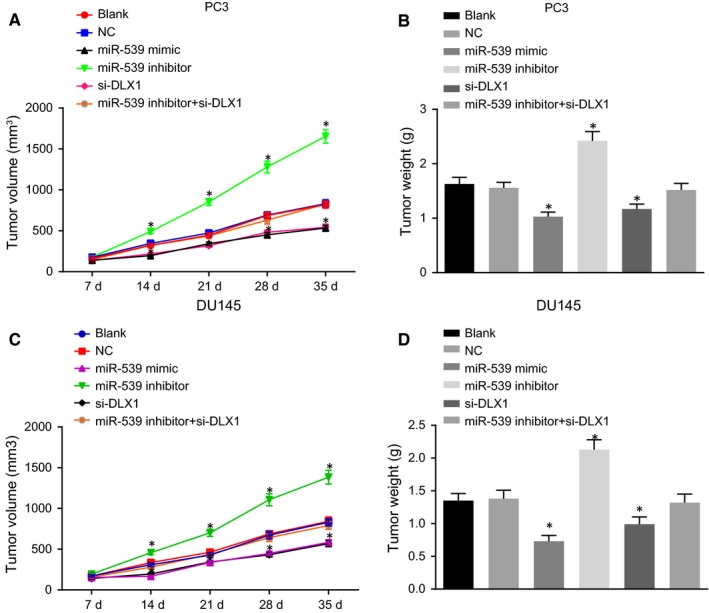
Up‐regulation of miR‐539 or silencing of DLX1 inhibits the tumour growth in nude mice. A and B, xenograft tumours and quantitative analysis of tumour mass of nude mice injected with PC3 cells in response to miR‐539 mimic, miR‐539 inhibitor, si‐DLX1 and miR‐539 inhibitor + si‐DLX1; C and D, xenograft tumours and quantitative analysis of tumour mass of nude mice injected with DU145 cells in response to miR‐539 mimic, miR‐539 inhibitor, si‐DLX1 and miR‐539 inhibitor + si‐DLX1; *P < 0.05, compared with the blank and NC groups; PCa, prostate cancer; miR‐539, microRNA‐539; NC, negative control; si, small interfering

Finally, in vivo tumour formation experiments in nude mice were conducted to further elucidate the effects of miR‐539 on PCa progression. As depicted in Figure 9, PC3 and DU145 lines showed the similar effects on the tumour growth of nude mice. There were no evident changes in relation to tumour growth between the blank and NC groups (*P* > 0.05). Compared with the blank and NC groups, the tumour growth was significantly decreased in the miR‐539 mimic group and the siRNA‐DLX1 group, while it was significantly elevated in the miR‐539 inhibitor group (*P* < 0.05) and the miR‐539 inhibitor + si‐DLX1 group presented with no significant difference (*P* > 0.05). In conclusion, miR‐539 overexpression or DLX1 silencing could result in the suppression of tumour growth rate in nude mice.

### 3.11 Up‐regulation of miR‐539 impedes PCa cell proliferation, migration and invasion through the inhibition of the TGF‐β signalling pathway

**Figure 10:**
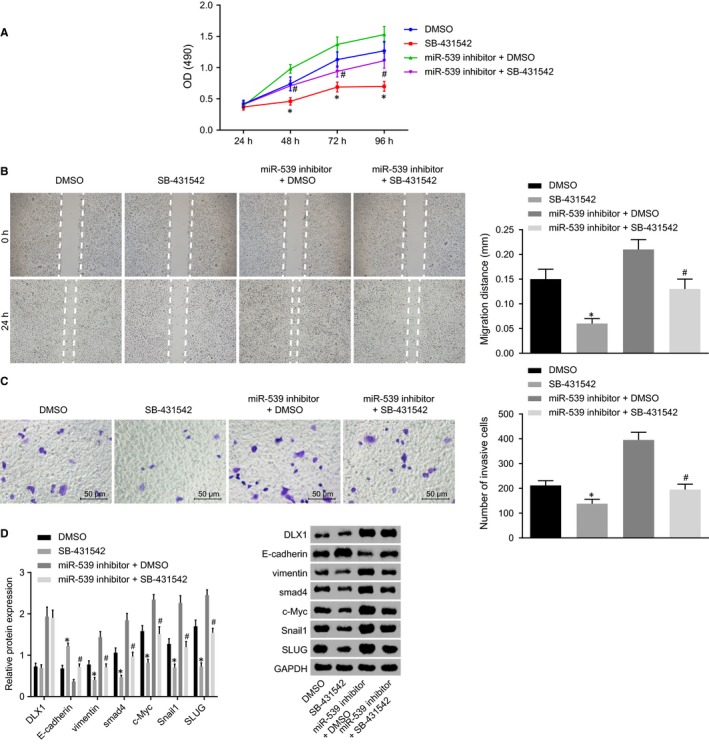
Up‐regulation of miR‐539 impedes PCa cell proliferation, migration and invasion through the inhibition of the TGF‐β signalling pathway. A, proliferation of DU145 cells transfected with SB‐431542, miR‐539 inhibitor, DMSO, miR‐539 inhibitor + DMSO and miR‐539 inhibitor + SB‐431542 measured by MTT assay; B, migration of DU145 cells transfected with SB‐431542, miR‐539 inhibitor, DMSO, miR‐539 inhibitor + DMSO and miR‐539 inhibitor + SB‐431542 measured by scratch test, C, invasion of DU145 cells transfected with SB‐431542, miR‐539 inhibitor, DMSO and miR‐539 inhibitor + SB‐431542 measured by transwell assay; D, Western blot analysis of vimentin, E‐cadherin, c‐Myc, Smad4, Snail1 and SLUG proteins in DU145 cell lines transfected with SB‐431542, miR‐539 inhibitor, DMSO, miR‐539 inhibitor + DMSO and miR‐539 inhibitor + SB‐431542; *P < 0.05, compared with the DMSO group; # *P* < 0.05, compared with the miR‐539 inhibitor + DMSO group

MTT assay, scratch test and transwell assay were conducted to elucidate the role of miR‐539 in PCa cell proliferation, migration and invasion through TGF‐β signalling pathway. As shown in Figure 10A‐C, compared with the DMSO group, PCa cell proliferation, migration and invasion were significantly decreased in the SB‐431542 group (*P* < 0.05). In comparison with the miR‐539 inhibitor + DMSO group, miR‐539 inhibitor + SB‐431542 group also presented with a significant decrease in cell proliferation, migration and invasion (*P* < 0.05). Western blot analysis revealed that SB‐431542 group had higher E‐cadherin protein level, yet lower levels of vimentin, Snail1, SLUG, c‐Myc and Smad4 protein when compared with the DMSO group (*P* < 0.05) (Figure 10D). In comparison with the miR‐539 inhibitor + DMSO group, E‐cadherin protein level was increased while protein levels of vimentin, Snail1, SLUG, c‐Myc and Smad4 were down‐regulated in the miR‐539 inhibitor + SB‐431542 group (*P* < 0.05). There were no significant changes observed in relation to DLX1 protein levels between the DMSO and SB‐431542 groups, the miR‐539 inhibitor + DMSO and miR‐539 inhibitor + SB‐431542 groups. The aforementioned results highly suggested that miR‐539 overexpression could potentially disrupt PCa cell proliferation, migration and invasion by inactivating the TGF‐β signalling pathway.

## 4 DISCUSSION

Various miRNAs have been demonstrated to modulate pathological processes of a variety of malignancies, including differentiation, migration, invasion and EMT of cancer cells.^3^, ^26^ The inhibitory role of miR‐539 in the progression of several types of cancer has been previously reported, including in breast cancer by targeting EGFR,^27^ in hepatocellular carcinoma by binding to FSCN1,^28^ and in colorectal cancer by directly binding to RUNX2.^29^ Based on these findings, we conducted the study with aims of determining the underlying effects of miR‐539 on EMT and metastasis of PCa, on the basis of miR‐539 being involved in the suppression of EMT, invasion, migration and invasion by inactivating the TGF‐β/Smad4 signalling pathway through the down‐regulation of DLX1 in PCa.

The initial findings of our study indicated that the there was a poor expression in miR‐539 while DLX1 was highly expressed in PCa tissues. Consistently, a previous study has also indicated that there is a significant down‐regulation in miR‐539 expression level in PCa tissues, thereby serving as a potential anti‐progression biomarker.^15^ In addition, the reduction in miR‐539 expression has also been detected in triple‐negative breast cancer and the up‐regulated miR‐539 could suppress tumour progression by negatively regulating and targeting the expression of LAMA4.^30^ The expression of miR‐539 was reported to be negatively linked to the lymph node metastasis and clinical phase of human colorectal cancer, suggesting that miR‐539 could slow the progression of colorectal cancer.^29^ Moreover, there is an overexpression in DLX1 in PCa and DLX1, which could be considered as an underlying marker for the treatment of PCa.^18^ There has also been a study indicating that DLX1 can serve as a promising indicator for high‐grade PCa detection.^31^ In addition to PCa, high‐grade serous ovarian cancer was also found to have presented with increased DLX1, indicating that it can also play a role as a therapeutic biomarker for high‐grade serous ovarian cancer.^22^

Furthermore, predication based on the bioinformatic website revealed that miR‐539 could target and result in the down‐regulation of DLX1 expression, which was confirmed by dual luciferase reporter gene assay. Consistently, miR‐539 was found to directly target SPAG5 and miR‐539 was negatively associated with that of SPAG5 in PCa.^15^ Furthermore, EGFR was verified to be targeted and down‐regulated by miR‐539 in breast cancer and the binding of miR‐539 to EGFR serves as a biomarker of the treatment of breast cancer.^27^

In addition, our findings also suggested that the study concluded that implementing the enhancement of miR‐539 expression can result in the suppression of proliferation, migration, invasion and EMT of PCa cells as evidenced by elevated expression of E‐cadherin and decreased expression of vimentin, c‐Myc following treatment with miR‐539. Similarly, by binding to CARMA1, miR‐539 leads to the suppression of cell migration and invasion in thyroid cancer.^32^ Consistently, the EMT, tumourigenicity and metastatic capacity of PCa cells could be inhibited through the overexpression of miR‐200b, as a result of the up‐regulated expression of E‐cadherin.^33^ The decrease in the level of vimentin has been proven to have an inhibitory effect on motility and invasion of PCa cells when administrated alongside antisense‐vimentin.^34^ Moreover, silibinin, which has been found to provide protection against PCa, has been suggested to suppress the progression of PCa through the down‐regulation of vimentin.^35^ c‐Myc, which is an oncogene, is involved in cancer progression and its overexpression is clearly associated with higher histological grade and unfavourable prognosis of patients with PCa.^36^

Our findings also demonstrated that miR‐539 resulted in the down‐regulation of Smad4, Snail1 and SLUG in PCa cells, indicating that miR‐539 could lead to the inhibition of the activation of the TGF‐β/Smad4 signalling pathway. As a critical regulator of the TGF‐β signalling pathway, Smad4 usually exerts tumour‐suppressive effects in numerous cancers, including PCa.^20^, ^37^ Aitchison et al have demonstrated that promoter methylation associates with decreased Smad4 expression in advanced PCa.^37^ Smad4 reduction could induce EMT in head and neck squamous cell carcinoma.^38^ A previously conducted investigation revealed that TGF‐β‐mediated invasion and metastasis of colorectal cancer cells can be promoted by succinate dehydrogenase deficiency through transcriptional repression complex Snail1‐Smad3/4.^39^ miR‐9 has been identified to up‐regulate E‐cadherin expression and result in significantly decreased melanoma cell proliferation and migration capacity through the inhibition of NF‐κB1‐Snail1 signaling pathway.^40^ SLUG is a zinc‐finger transcription factor of the Snail/SLUG zinc‐finger family that plays a role in migration and invasion of tumour cells.^41^ An increase in SLUG expression has been detected in advanced‐stage primary PCa and the knock‐down of SLUG results in the impairment of migration and invasion of PCa cells.^42^ Consistently, the inactivation of the TGF‐β/Smad2/3 signalling pathway was found to inhibit metastasis and EMT in PCa.^43^ miR‐155 has been found to play a functional role in EMT, cell migration and invasion induced by the TGF‐β signalling pathway in invasive breast cancer.^44^ Collectively, miR‐539 inhibited EMT, invasion and migration by inhibiting the TGF‐β/Smad4 signalling pathway.

## 5 CONCLUSION

**Figure 11:**
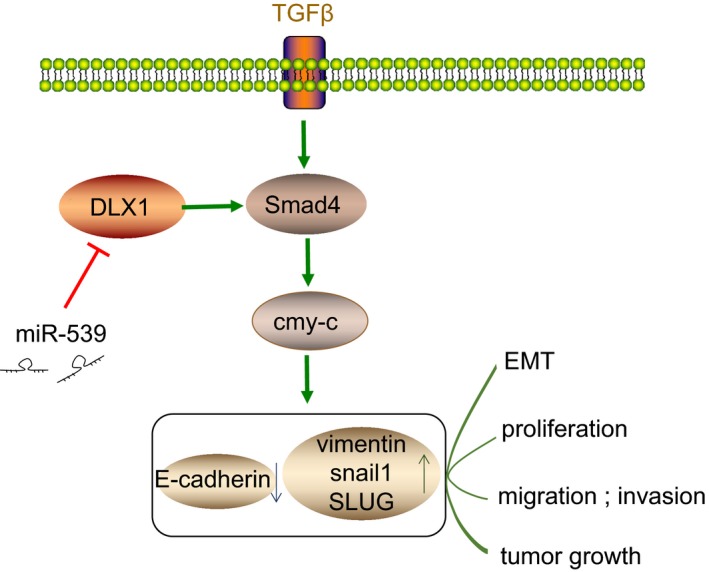
Schematic representation of inhibitory effect of miR‐539 on EMT and metastasis of PCa. miR‐539 could down‐regulate DLX1 expression, thus elevating the expression of E‐cadherin but decreasing the expression of DLX1, vimentin, c‐Myc and Smad4. These alterations led to inhibited proliferation, migration, invasion and tumour growth of PCa cells. miR‐539, microRNA‐539; PCa, prostate cancer; DLX1, distal‐less homeobox 1; EMT, epithelial‐mesenchymal transition

In conclusion, the aforementioned findings provided evidence that miR‐539 could result in the suppression of cell migration, invasion, migration and EMT in PCa by inactivating the TGF‐β/Smad4 signalling pathway through the direct down‐regulation of DLX1 (Figure 11), which provides new insights for future study of PCa treatment. However, this study is still at the pre‐clinical stage and the investigation on the mechanism of action is not yet well elucidated. Therefore, further large‐scale studies are required to broaden the therapeutic perspective of PCa from the cellular level by understanding cell capacity and cell‐cell interaction.

## CONFLICT OF INTEREST

None declared.

## AUTHOR CONTRIBUTION

Baogang Sun and Yingying Fan participated in the conception and design of the study. Aijun Yang and Lunan Liang performed the analysis and interpretation of data. Baogang Sun and Jinghe Cao contributed to drafting the article. All authors participated in the revised manuscript and have read and approved the final submitted manuscript.

## References

[1] Jemal A , Bray F , Center MM , Ferlay J , Ward E , Forman D . Global cancer statistics. CA Cancer J Clin. 2011;61:69‐90. 21296855 10.3322/caac.20107

[2] Shao Q , Ouyang J , Fan Y , et al. Prostate cancer in the senior men from rural areas in east district of China: contemporary management and 5‐year outcomes at multi‐institutional collaboration. Cancer Lett. 2012;315:170‐177. 22099876 10.1016/j.canlet.2011.09.035

[3] Khan MI , Hamid A , Adhami VM , et al. Role of epithelial mesenchymal transition in prostate tumorigenesis. Curr Pharm Des. 2015;21:1240‐1248. 25506896 10.2174/1381612821666141211120326 PMC4362522

[4] Watahiki A , Wang Y , Morris J , et al. MicroRNAs associated with metastatic prostate cancer. PLoS ONE. 2011;6:e24950. 21980368 10.1371/journal.pone.0024950 PMC3184096

[5] Chen L , Mai W , Chen M , et al. Arenobufagin inhibits prostate cancer epithelial‐mesenchymal transition and metastasis by down‐regulating beta‐catenin. Pharmacol Res. 2017;123:130‐142. 28712972 10.1016/j.phrs.2017.07.009

[6] Ru P , Steele R , Newhall P , Phillips NJ , Toth K , Ray RB . miRNA‐29b suppresses prostate cancer metastasis by regulating epithelial‐mesenchymal transition signaling. Mol Cancer Ther. 2012;11:1166‐1173. 22402125 10.1158/1535-7163.MCT-12-0100

[7] Liu C , Kelnar K , Liu B , et al. The microRNA miR‐34a inhibits prostate cancer stem cells and metastasis by directly repressing CD44. Nat Med. 2011;17:211‐215. 21240262 10.1038/nm.2284 PMC3076220

[8] Zhang H‐L , Yang L‐F , Zhu Y , et al. Serum miRNA‐21: elevated levels in patients with metastatic hormone‐refractory prostate cancer and potential predictive factor for the efficacy of docetaxel‐based chemotherapy. Prostate. 2011;71:326‐331. 20842666 10.1002/pros.21246

[9] Spahn M , Kneitz S , Scholz CJ , et al. Expression of microRNA‐221 is progressively reduced in aggressive prostate cancer and metastasis and predicts clinical recurrence. Int J Cancer. 2010;127:394‐403. 19585579 10.1002/ijc.24715

[10] Bracken CP , Scott HS , Goodall GJ . A network‐biology perspective of microRNA function and dysfunction in cancer. Nat Rev Genet. 2016;17:719‐732. 27795564 10.1038/nrg.2016.134

[11] Harazono Y , Muramatsu T , Endo H , et al. miR‐655 Is an EMT‐suppressive microRNA targeting ZEB1 and TGFBR2. PLoS ONE. 2013;8:e62757. 23690952 10.1371/journal.pone.0062757 PMC3653886

[12] Li B‐L , Lu C , Lu W , et al. miR‐130b is an EMT‐related microRNA that targets DICER1 for aggression in endometrial cancer. Med Oncol. 2013;30:484. 23392577 10.1007/s12032-013-0484-0

[13] Liu T , Nie F , Yang X , et al. MicroRNA‐590 is an EMT‐suppressive microRNA involved in the TGFbeta signaling pathway. Mol Med Rep. 2015;12:7403‐7411. 26459119 10.3892/mmr.2015.4374 PMC4626157

[14] Cao Z , Zheng X , Cao L , Liang N . MicroRNA‐539 inhibits the epithelial‐mesenchymal transition of esophageal cancer cells by twist‐related protein 1‐Mediated modulation of melanoma‐associated antigen A4. Oncol Res. 2018;26:529‐536. 28653599 10.3727/096504017X14972679378357 PMC7844688

[15] Zhang H , Li S , Yang X , Qiao B , Zhang Z , Xu Y . miR‐539 inhibits prostate cancer progression by directly targeting SPAG5. J Exp Clin Cancer Res. 2016;35:60. 27037000 10.1186/s13046-016-0337-8 PMC4818461

[16] McDougall C , Korchagina N , Tobin JL , Ferrier D . Annelid Distal‐less/Dlx duplications reveal varied post‐duplication fates. BMC Evol Biol. 2011;11:241. 21846345 10.1186/1471-2148-11-241 PMC3199776

[17] Alinezhad S , Väänänen R‐M , Mattsson J , et al. Validation of novel biomarkers for prostate cancer progression by the combination of bioinformatics, Clinical and Functional Studies. PLoS ONE. 2016;11:e0155901. 27196083 10.1371/journal.pone.0155901 PMC4873225

[18] Liang M , Sun Y , Yang H‐L , Zhang B , Wen JI , Shi B‐K . DLX1, a binding protein of beta‐catenin, promoted the growth and migration of prostate cancer cells. Exp Cell Res. 2018;363:26‐32. 29317218 10.1016/j.yexcr.2018.01.007

[19] Pu H , Horbinski C , Hensley PJ , Matuszak EA , Atkinson T , Kyprianou N . PARP‐1 regulates epithelial‐mesenchymal transition (EMT) in prostate tumorigenesis. Carcinogenesis. 2014;35:2592‐2601. 25173886 10.1093/carcin/bgu183 PMC4216059

[20] Demagny H , De Robertis EM . Point mutations in the tumor suppressor Smad4/DPC4 enhance its phosphorylation by GSK3 and reversibly inactivate TGF‐beta signaling. Mol Cell Oncol. 2016;3:e1025181. 27308538 10.1080/23723556.2015.1025181 PMC4845174

[21] Fu H , He H‐C , Han Z‐D , et al. MicroRNA‐224 and its target CAMKK2 synergistically influence tumor progression and patient prognosis in prostate cancer. Tumour Biol. 2015;36:1983‐1991. 25394900 10.1007/s13277-014-2805-0

[22] Chan DW , Hui WW , Wang JJ , et al. DLX1 acts as a crucial target of FOXM1 to promote ovarian cancer aggressiveness by enhancing TGF‐beta/SMAD4 signaling. Oncogene. 2017;36:1404‐1416. 27593933 10.1038/onc.2016.307 PMC5348575

[23] Chiba S , Takeshita K , Imai Y , et al. Homeoprotein DLX‐1 interacts with Smad4 and blocks a signaling pathway from activin A in hematopoietic cells. Proc Natl Acad Sci USA. 2003;100:15577‐15582. 14671321 10.1073/pnas.2536757100 PMC307610

[24] Wa Q , Li L , Lin H , et al. Downregulation of miR19a3p promotes invasion, migration and bone metastasis via activating TGFbeta signaling in prostate cancer. Oncol Rep. 2018;39:81‐90. 29138858 10.3892/or.2017.6096 PMC5783607

[25] Valkenburg KC , De Marzo AM , Williams BO . Deletion of tumor suppressors adenomatous polyposis coli and Smad4 in murine luminal epithelial cells causes invasive prostate cancer and loss of androgen receptor expression. Oncotarget. 2017;8:80265‐80277. 29113300 10.18632/oncotarget.17919 PMC5655195

[26] Lamouille S , Subramanyam D , Blelloch R , Derynck R . Regulation of epithelial‐mesenchymal and mesenchymal‐epithelial transitions by microRNAs. Curr Opin Cell Biol. 2013;25:200‐207. 23434068 10.1016/j.ceb.2013.01.008 PMC4240277

[27] Guo J , Gong G , Zhang B . miR‐539 acts as a tumor suppressor by targeting epidermal growth factor receptor in breast cancer. Sci Rep. 2018;8:2073. 29391441 10.1038/s41598-018-20431-z PMC5794864

[28] Liu Y , Hong W , Zhou C , et al. miR‐539 inhibits FSCN1 expression and suppresses hepatocellular carcinoma migration and invasion. Oncol Rep. 2017;37:2593‐2602. 28393215 10.3892/or.2017.5549 PMC5428223

[29] Wen D , Li S , Jiang W , Zhu J , Liu J , Zhao S . miR‐539 inhibits human colorectal cancer progression by targeting RUNX2. Biomed Pharmacother. 2017;95:1314‐1320. 28938522 10.1016/j.biopha.2017.09.044

[30] Yang Z‐X , Zhang BO , Wei J , et al. MiR‐539 inhibits proliferation and migration of triple‐negative breast cancer cells by down‐regulating LAMA4 expression. Cancer Cell Int. 2018;18:16. 29434522 10.1186/s12935-018-0512-4 PMC5791727

[31] Van Neste L , Hendriks RJ , Dijkstra S , et al. Detection of high‐grade prostate cancer using a urinary molecular biomarker‐based risk score. Eur Urol. 2016;70:740‐748. 27108162 10.1016/j.eururo.2016.04.012

[32] Gu L , Sun W . MiR‐539 inhibits thyroid cancer cell migration and invasion by directly targeting CARMA1. Biochem Biophys Res Commun. 2015;464:1128‐1133. 26206083 10.1016/j.bbrc.2015.07.090

[33] Williams LV , Veliceasa D , Vinokour E , Volpert OV . miR‐200b inhibits prostate cancer EMT, growth and metastasis. PLoS ONE. 2013;8:e83991. 24391862 10.1371/journal.pone.0083991 PMC3877136

[34] Zhao Y , Yan Q , Long X , Chen X , Wang Y . Vimentin affects the mobility and invasiveness of prostate cancer cells. Cell Biochem Funct. 2008;26:571‐577. 18464297 10.1002/cbf.1478

[35] Wu K‐J , Zeng J , Zhu G‐D , et al. Silibinin inhibits prostate cancer invasion, motility and migration by suppressing vimentin and MMP‐2 expression. Acta Pharmacol Sin. 2009;30:1162‐1168. 19578386 10.1038/aps.2009.94 PMC4006687

[36] Mao A , Zhao Q , Zhou X , et al. MicroRNA‐449a enhances radiosensitivity by downregulation of c‐Myc in prostate cancer cells. Sci Rep. 2016;6:27346. 27250340 10.1038/srep27346 PMC4890029

[37] Aitchison AA , Veerakumarasivam A , Vias M , et al. Promoter methylation correlates with reduced Smad4 expression in advanced prostate cancer. Prostate. 2008;68:661‐674. 18213629 10.1002/pros.20730

[38] Cheng H , Fertig EJ , Ozawa H , et al. Decreased SMAD4 expression is associated with induction of epithelial‐to‐mesenchymal transition and cetuximab resistance in head and neck squamous cell carcinoma. Cancer Biol Ther. 2015;16:1252‐1258. 26046389 10.1080/15384047.2015.1056418 PMC4623002

[39] Wang H , Chen Y , Wu G . SDHB deficiency promotes TGFbeta‐mediated invasion and metastasis of colorectal cancer through transcriptional repression complex SNAIL1‐SMAD3/4. Transl Oncol. 2016;9:512‐520. 27816688 10.1016/j.tranon.2016.09.009 PMC5097976

[40] Liu S , Kumar SM , Lu H , et al. MicroRNA‐9 up‐regulates E‐cadherin through inhibition of NF‐kappaB1‐Snail1 pathway in melanoma. J Pathol. 2012;226:61‐72. 22131135 10.1002/path.2964 PMC3959162

[41] Uygur B , Wu WS . SLUG promotes prostate cancer cell migration and invasion via CXCR41/CXCL12 axis. Mol Cancer. 2011;10:139. 22074556 10.1186/1476-4598-10-139 PMC3226635

[42] Liu YN , Abou‐Kheir W , Yin JJ , et al. Critical and reciprocal regulation of KLF4 and SLUG in transforming growth factor beta‐initiated prostate cancer epithelial‐mesenchymal transition. Mol Cell Biol. 2012;32:941‐953. 22203039 10.1128/MCB.06306-11 PMC3295188

[43] Kou B , Liu W , Zhao W , et al. Thymoquinone inhibits epithelial‐mesenchymal transition in prostate cancer cells by negatively regulating the TGF‐beta/Smad2/3 signaling pathway. Oncol Rep. 2017;38:3592‐3598. 29039572 10.3892/or.2017.6012

[44] Kong W , Yang H , He L , et al. MicroRNA‐155 is regulated by the transforming growth factor beta/Smad pathway and contributes to epithelial cell plasticity by targeting RhoA. Mol Cell Biol. 2008;28:6773‐6784. 18794355 10.1128/MCB.00941-08 PMC2573297

